# 

**Published:** 1915-11-13

**Authors:** 


Dr. A. H. Blunt has received the sad news that his second son, Second-Lieutenant Francis Clifford Blunt, of the 4th Leicesters, had died from wounds on October 14. The deceased officer joined the Territorials after the outbreak of war, and on the completion of his training went to the Front. He was home again about Easter, when he received a commission, and subsequently returned to France.

### Death of a Local Government Board Inspector.

By the death of Dr. Brian O'Brien the Local Government Board in Ireland loses one of its medical inspectors, and by the irony of fate he is carried off by the very disease which has caused so much anxiety to the Board, especially in Belfast. On October 29 Dr. O'Brien died of cerebro-spinal meningitis in the Pardyburn Hospital at Belfast. He qualified at the University of Dublin in 1896, and took his M.D. degree two years later.

### Treatment.

### Personalia.

Mr. J. H. Johnson, secretary of the Royal Westminster Ophthalmic Hospital, King William Street, West Strand, London, W.C., has accepted an organising appointment from the War Office, with rank of lieutenant, in the Army Service Corps.

The newly elected president of the Association of Registered Medical Women is Dr. A. Helen A. Boyle, M.D. (Brux.), L.R.C.P., L.R.C.S. (Edin.). Last April Dr. Boyle went out to Serbia to join Mr. and Mrs. James Berry's unit at Vrujatchka Banja, taking with her two women orderlies and a large Thresh disinfector and destructor. At the annual meeting of the Association, held last month, she gave an exceptionally interesting account of some of her experiences in fever and gynæcological work in Serbia.

### Buildings Contemplated.

Belfast.—The new wing added to the U.V.F. Hospital, Botanic Avenue, has been formally opened. Mr. R. J. Calwell, of Belfast, was the architect, and Mr. W. Dowling the contractor.

Eccles.—Extensions are to be carried out at the Elm Bank Hospital.

Pontypool.—The new Nurses' Home at the Pontypool and District Hospital has been formally opened. The architects were Messrs. D. J. Lougher and Co., of Pontypool, and the builders Messrs. J. Evans and Co., of Pontnewynydd.

Skipton.—The foundation-stone of a new wing to the Skipton and District Hospital has been formally laid.

Swansea.—The building of the proposed tuberculosis hospital and the isolation hospital adjoining will proceed as soon as the war is over.

### Death of a Dublin Physician.

One of the most prominent physicians in Dublin has just passed away in the person of Dr. James B. Coleman, C.M.G., F.R.C.P.I. As physician to the Richmond Hospital since 1896 he was greatly admired by the crowds of students who eagerly attended his classes. He undertook an immense amount of research work on tuberculin and the opsonic method at the National Hospital for Consumption at Newcastle, Wicklow, to which he was appointed one of the two visiting physicians from the time of its opening in 1896. Dr. Coleman received the C.M.G. and a medal, with three clasps, for his work in 1900 as chief physician to Lord Iveagh's Irish Field Hospital, in which capacity he worked strenuously, particularly on the treatment of typhoid fever. Not many months ago he accepted an invitation to take charge of the medical side of a base hospital at the Front.

### PASSING EVENTS AND TOPICS.

### Not "At Home" on Sundays.

The Lancashire Insurance Committee have been asked on behalf of local practitioners to adopt a rule that an insured person shall not call upon the services of a panel doctor on Sundays except in cases of emergency.

### Casualties in the German Medical Service.

According to the *Deutsche Militäräztliche Zeitschrift*, the number of casualties in the Medical Corps of the German Army and Navy for the first thirteen months of the war was as follows: Wounded, 437; missing, 125; captured, 95; killed, 119; died of wounds, 45; died of disease, 398; killed by accident, 20. Total, 1,239.

### Medical Students and the War.

We have received the following official notice:—"The President of the General Medical Council is requested by the Council to inform the licensing bodies, medical schools, and approved teaching institutions that the Director-General of the Army Medical Service has intimated to the Council his entire agreement with the Earl of Derby's decision regarding the recruiting of medical students—namely, that 'It is the duty of medical students (other than those in the fourth and fifth years of study) to join His Majesty's Forces.' The President hopes that in every medical school steps will be taken to convey this information to the students who are eligible for military service."

### Gas Poisoning in Action.

To the *Journal of the Royal Army Medical Corps* Lieutenants Black, Glenny, and McNee contribute their experience in treating cases of gas poisoning caused by the enemy's adoption of this mode of warfare. They divide their cases into (1) the acute asphyxial, and (2) the subacute kinds. Of the former almost a quarter die; orthopnœa and cyanosis are the most characteristic signs, and treatment is of little avail. The subacute cases respond well to treatment; ammonium carbonate in full and frequent doses appears to have given the best results, and pituitary extract was also useful; atropine was somewhat disappointing.

### The Gait of Men who have Lost Limbs.

In a paper read before the Paris Academy of Medicine, Dr. Ducroquet stated the results of his observations of the gait of men provided with an artificial leg. He has drawn from these observations conclusions which may be of considerable value, and has made a comparison between the two types of artificial legs, the French and the American. The American appliance permits free flexion of the knee, and the gait with such a leg is better, but more fatiguing. In cases of amputation of the lower leg, whether the appliance be French or American, the gait, though different in the two cases, is often so perfect that the amputation is not suspected.

### An Electro-Vibratory Localiser.

A comparatively new localiser for ascertaining the presence of bullets or pieces of metal in the body is now being used in many French hospitals. This electro-vibratory localiser is associated with the name of Dr. Bergonié, and consists of an iron cone connected with the source of an alternating current. If the cone be held a few inches from the body and the hand passed over the skin, vibrations are at once felt over the position of any piece of metal, however small. Strictly speaking, the information thus gained refers only to the presence of a piece of metal and not to its exact localisation in the tissues, but the method is so simple that this apparatus must be accounted a distinct gain to military surgery.

### Injuries in Aviation Accidents.

The surgeon to a German aviation corps, who has had opportunities of observing injuries sustained by falling from a great height, reports—according to the Berlin correspondent of the official organ of the American Medical Association—that as a rule there are found fractures of the base of the skull, tearing of the lung tissues, and typical fractures of the upper and lower extremities, no matter how far the body has fallen. Fracture of the sternal end of the clavicle, of the forearm, of the lower leg above the epiphyseal line, and in the middle of the femur in the upper third, and complete comminution of the tarsus are present in nearly every case. Injuries of the spinal column are seen but rarely, whereas the viscera frequently burst or are torn to pieces; especially is this true of the heart, liver, lungs, and the large blood-vessels.

### The Latest Extension of St. Thomas's Hospital.

The current issue of *St. Thomas's Hospital Gazette* contains an interesting letter from somewhere in France describing what is termed "the latest extension of St. Thomas's Hospital." The 2nd Rifle Brigade and the 1st Royal Irish Rifles in turn hold the same trenches, and between them they have built a huge dug-out for use as a dressing station during shell fire, and over the door placed a board bearing the legend "St. Thomas's Hospital." The German aeroplanes apparently spotted it in the course of construction and their guns put ninety-two 4.2 in. shells in the immediate neighbourhood during the course of a single morning, but "St. Thomas's" was only slightly damaged. The medical officer of the 2nd Rifle Brigade, by the way, is Lieut. J. C. Maclean, M.D., an old St. Thomas's man, who has lately won the Military Cross.

RATES OF SUBSCRIPTION: Twelve months, 6/6; Six months, 4/-; Three months, 2/-, post free to any address in the United Kingdom. Abroad, Twelve months, 8/8; Six months, 5/-; Three months, 2/6, post free.—Address The Subscription Dept., "THE HOSPITAL," The Hospital Building, 28/29 Southampton Street, Strand, London, W.C.

